# The impact of renal insufficiency and anaemia on survival in patients with cardiovascular disease: a cohort study

**DOI:** 10.1186/1471-2261-9-51

**Published:** 2009-11-12

**Authors:** Jocelyn Anderson, Liam G Glynn, John Newell, Alberto A Iglesias, Donal Reddan, Andrew W Murphy

**Affiliations:** 1 Department of General Practice, NUI Galway, Ireland; 2 Department of General Practice, NUI Galway, Ireland; 3 Clinical Research Facility, NUI Galway, Ireland; 4 Dept of Mathematics, NUI Galway, Ireland; 5 Division of Nephrology, Dept of Medicine, NUI Galway, Ireland; 6 Department of General Practice, NUI Galway, Ireland

## Abstract

**Background:**

The simultaneous occurrence of cardiovascular disease (CVD), kidney disease, and anaemia is associated with increased morbidity and mortality. In the community setting, little data exists about the risk associated with milder levels of anaemia when it is present concurrently with CVD and chronic kidney disease (CKD). The aim of this study was to establish the prevalence of CKD and anaemia in patients with CVD in the community and to examine whether the presence of anaemia was associated with increased morbidity and mortality.

**Methods:**

This study was designed as a retrospective cohort study and involved a random sample of 35 general practices in the West of Ireland. A practice-based sample of 1,609 patients with established cardiovascular disease was generated in 2000/2001 and followed for five years. The primary endpoint was death from any cause. Statistical analysis involved using one-way ANOVA and Chi-squared tests for baseline data and Cox proportional-hazards models for mortality data.

**Results:**

Of the study sample of 617 patients with blood results, 33% (n = 203) had CKD while 6% (n = 37) had CKD and anaemia. The estimated risk of death from any cause, when compared to patients with cardiovascular disease only, was almost double (HR = 1.98, 95% CI 0.99 to 3.98) for patients with both CVD and CKD and was over 4 times greater (HR = 4.33, 95% CI 1.76 to 10.68) for patients with CVD, CKD and anaemia.

**Conclusion:**

In patients with cardiovascular disease in the community, chronic kidney disease and anaemia occur commonly. The presence of chronic kidney disease carries an increased mortality risk which increases in an additive way with the addition of anaemia. These results suggest that early primary care diagnosis and management of this high risk group may be worthwhile.

## Background

Chronic kidney disease (CKD) and cardiovascular disease (CVD) often coexist and have been known to exert a bidirectional effect on one another [1-4]. CKD is an established risk factor for the development of CVD and in patients with established cardiovascular disease, chronic kidney disease is associated with a significantly increased risk of cardiovascular mortality and morbidity [5-9]. The suspected etiologic pathways behind the cardio-renal interaction involve positive feedback loops between "cardiorenal connectors" including (but not limited to) sympathetic stimulation, the renin-angiotensin-aldosterone system and cytokines [10]. Anaemia, a condition frequently encountered in chronic disease, is an additional mediator in the progression of either CKD and/or CVD and it is also an independent risk factor for the onset of cardiovascular complications [11]. The prevalence of anaemia has been reported to occur in up to 50% of CKD patients [12] and 51% of heart failure patients [13] though results will vary depending on the setting, classification of anaemia and severity of disease. The interrelations between CKD, CVD, and anaemia are complex, poorly understood, and are referred to in the literature as the cardiorenal anaemia syndrome [14]. Each morbidity of the cardiorenal anaemia syndrome is said to independently exert an effect by causing or worsening the other mediators of the triad increasing the risk of adverse outcomes including death [1,2].

Though the three bi-directional relationships between CKD, anaemia and heart failure have been established, the majority of this research has focused on the hospitalized population and in patients with recognized congestive heart failure. While evidence for patients with advanced CKD participating in renal replacement therapy programs is quite robust, there appears to be little evidence in the community population regarding the relative and cumulative effect on prognosis of cardiovascular disease, chronic kidney disease and anaemia. Descriptions of prognosis derived from secondary care may be misleading, due to selection biases, when managing patients in primary care [13]. Others have previously shown that patients entered into hospital-based trials of blood pressure control after stroke have important age, gender and blood pressure differences to patients managed in the community after their stroke [15].

We utilised a cohort of patients, with the full spectrum of cardiovascular disease, from a random sample of practices in the community to establish the prevalence of CKD and anaemia and to examine whether the presence of CKD and anaemia was associated with increased mortality in patients with cardiovascular disease.

## Methods

### Study population and measurement of renal function

The study sample consisted of a cohort of 1,609 patients with cardiovascular disease who were identified from a stratified random sample of 35 general practices in the West of Ireland [16]. In order to generate this sample, practices were randomly selected, after stratification by practice type (single-handed or group) and location (rural or urban), from the Health Services Executive Western area and asked to participate in the study. Thirty five (60%) practices chose to do so and these practices were asked to generate a list of all their patients with established cardiovascular disease using multiple methods including practice disease registers, patient database searches, prescribing records, prospective recording of patient attendance and opportunistic practitioner recall. Patients were defined as having cardiovascular disease if they had a history of myocardial infarction (MI); angina; or revascularisation by percutaneous coronary intervention or cardiovascular bypass grafting. Patients were included in the current study where data were available on haemoglobin and creatinine status (serum sample measurement from the regional laboratory within the study period or up to 12 months prior to recruitment). Follow-up data on the cohort were collected after a period of five years and patients not experiencing events were censored at this point. Data on subjects were also censored where follow-up data ceased to be available. Renal function was assessed using estimated glomerular filtration rate (eGFR) calculated by the abbreviated Modified Diet in Renal Disease (MDRD) equation [17]. Patients were defined as having chronic kidney disease if they had an eGFR of <60 ml/min/1.73 m^2 ^according to National Kidney Foundation guidelines [18]. Anaemia status was assessed using full blood count data. Patients were define, according to WHO guideline, as having anaemia with a haemoglobin of <12.0 g/dl for females and <13.0 g/dl for males [19].

### Outcomes

The primary endpoint was death from any cause. Mortality data were collected from a search of practice records and the General Register Office, which is the central civil repository for records relating to Births, Marriages and Deaths in the Republic of Ireland.

### Statistical Analysis

For analysis of baseline characteristics and survival analysis, patients were classified according to presence of chronic kidney disease and anaemia. Baseline characteristics were analysed with the use of one-way ANOVA for continuous variables and Chi-square or Fisher's Exact test for categorical variables. Patient variables included 18 baseline characteristics (Table 1). To control for the large number of covariates, variable selection techniques were used to identify the most parsimonious model containing significant explanatory variables while including the covariate of interest (presence of chronic kidney disease and anaemia). Cox proportional-hazards models [Backwards elimination (with *Wald criterion*)] were used to evaluate the prognostic effect of presence of chronic kidney disease and anaemia over the study period while controlling for all explanatory variables. Adjusted hazard ratios, categorized according to level of multimorbidity, for death from any cause were determined while adjusting for: age, gender, GMS status, smoking status, systolic blood pressure, diastolic blood pressure, total cholesterol level, previous myocardial infarction, angina, heart failure, stroke, peripheral vascular disease, thromboembolic events, prior percutaneous coronary intervention, prior coronary artery bypass grafting, use of aspirin, B-Blocker, Lipid-lowering agent or ACE inhibitor. The assumptions underlying the final models were checked using suitable residual plots and interactions between presence of chronic kidney disease and anaemia and age and gender were investigated. All statistical test values were two-sided, and a p value of less than 0.05 was considered to indicate statistical significance. Analysis was carried out using SPSS (15.0) and *R *statistical software.

**Table 1:** Baseline characteristics of 617 patients with cardiovascular disease according to level of cardiovascular multimorbidity

Characteristic	CVD only (n = 339)	CVD & CKD (n = 203)	CVD & Anaemia (n = 38)	CVD & CKD & Anaemia (n = 37)	p value*
**Mean eGFR (ml/min/1.73 m^2^) (SD)**	74.47 (13.4)	51.18 (10.3)	72.08 (11.6)	45.81 (14.5)	**<0.01**

**Mean Hb (g/dL) (SD)**	14.50 (1.11)	13.95 (1.14)	11.13 (1.57)	11.05 (1.53)	**<0.01**

**Mean Age in years (SD)**	64.49 (8.9)	69.74 (7.48)	66.90 (9.62)	72.64 (4.99)	**<0.01**

**Female sex (%)**	27.4	60.3	39.5	63.1	**<0.01**

**Current Smoker (%)**	30.4	24.8	40.0	30.8	0.676

**Previous CVD event (%)**					

MI	46.6	41.1	44.7	55.3	0.361

Angina	83.7	88.7	86.8	81.6	0.379

Heart Failure	8.0	11.8	7.9	10.5	0.502

**Previous Co-morbidity (%)**					

Peripheral vascular disease	5.0	7.8	5.3	10.5	0.347

Stroke	4.4	5.9	2.6	0.0	0.481

Thromboembolism(PE, DVT, TIA)	10.3	18.1	2.6	10.5	**0.012**

**Baseline Clinical status (SD)**					

Systolic blood pressure (mm Hg)	137.2 (17.7)	138.8 (19.8)	133.8 (15.5)	139.6 (21.3)	0.418

Diastolic blood pressure (mm Hg)	80.6 (9.3)	79.9 (8.7)	77.2 (10.0)	81.2 (8.4)	0.172

Total Cholesterol (mmol/L)	5.3 (0.97)	5.4 (1.0)	4.9 (0.9)	5.5 (1.4)	**0.031**

**Baseline medication (%)**					

Aspirin	75.4	67.5	71.0	81.6	0.131

B-Blocker	51.0	46.3	55.3	34.2	0.170

Lipid-lowering agent	50.7	50.7	36.8	28.9	**0.029**

ACE Inhibitor	25.2	25.1	26.3	31.6	0.854

*p value refers to the one-way ANOVA and Chi-squared analyses comparing the 4 groups

## Results

### Baseline Characteristics

Among the original 1,609 patients in the study, 42 (2.6%) patients were lost to follow-up and 617 (38%) had complete blood data. Median follow-up was 4.50 years (SD 0.4) and there was no significant difference in age, gender, social status, smoking status, diabetic status and previous cardiovascular morbidity between those patients with, and without, complete blood data. Table 1 describes the baseline characteristics of the patients according to presence of chronic kidney disease and anaemia. A total of 339 (54.9%) patients had cardiovascular disease only; 203 (32.9%) had cardiovascular disease and chronic kidney disease; 38 (6.1%) patients had cardiovascular disease and anaemia while 37 (6.0%) patients had cardiovascular disease, chronic kidney disease and anaemia. Patients with multimorbidity involving all three conditions were significantly older, more likely to be female, have the lowest mean eGFR and haemoglobin (Hb) levels, the highest total cholesterol levels and were least likely to be taking a lipid lowering agent when compared with the other patient groups with fewer co-morbidities.

### Outcomes

**Figure 1:**
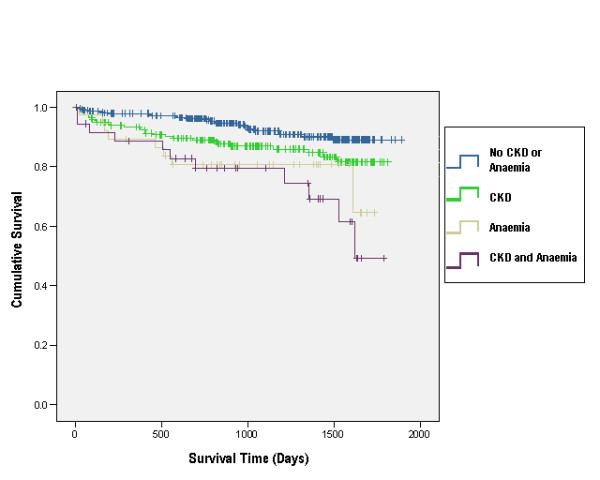
**Kaplan-Meier survival plots for risk of death in patients with cardiovascular disease**. (see attached file). Kaplan-Meier survival plot for risk of "death from any cause" in patients with cardiovascular disease in the community. Results are shown according to presence of CKD and/or anaemia.

During follow-up there were 72 deaths and 90 cardiovascular events. The risk of death from any cause [Log Rank (Mantel-Cox) 22.08, p < 0.001] was significantly increased in those patients with increased level of multimorbidity (Table 2, Figure 1). In the examination of the relationship between the primary outcome and presence of chronic kidney disease and anaemia, patients with cardiovascular disease only ['CVD only'] were used as the reference group. The adjusted hazard ratios (HR) for mortality according to presence of chronic kidney disease and anaemia are displayed in Table 3. The estimated risk of death from any cause, when compared to patients with cardiovascular disease only, was almost double (HR = 1.98, 95% CI 0.99 to 3.98) for patients with both cardiovascular disease and chronic kidney disease and was over 4 times greater (HR = 4.33, 95% CI 1.76 to 10.68) for patients with cardiovascular disease, chronic kidney disease and anaemia. No significant two-way interactions were identified between multimorbidity and other factors in the model when considering death by any cause.

**Table 2:** Kaplan-Meier summary statistics for risk of death^a ^according to presence of CKD and anaemia Case Processing Summary

CKD Anaemia Multimorbidity	Total N	N of Events	Censored	
	**N**	**Percent**	**N**	**Percent**

No CKD or Anaemia	339	25%	314	92.6%
CKD	203	28%	175	86.2%
Anaemia	38	8%	30	78.9%
CKD + Anaemia	37	11%	26	70.3%
Overall	617	72%	545	88.3%

**Overall Comparisons**				
	
	**Chi-Square**	**df**	**Sig**.	
	
Log Rank (Mantel-Cox)	22.083	3	.000	

Test of equality of survival distributions for the different levels of CKD Anaemia Multimorbidity.

^a^Risk of "death from any cause"

**Table 3:** ^a^Adjusted hazard ratios for death endpoint among patients according to presence of anaemia and CKD

Classification	Death from any cause	
	
		**exp**()**((95% C.I.)**
CVD only		**1.00**

CVD + CKD	**.685**	**1.985 (0.990,3.980)**

CVD + Anaemia	**.857**	**2.356 (0.780,7.114)**

CVD + CKD + Anaemia	**1.467**	**4.334 (1.759,10.681)**

Total number of patients = 617.

^a^The Cox proportional hazards model used in the above analysis adjusted for the following covariates: age, gender, GMS status, smoking status, systolic blood pressure, diastolic blood pressure, total cholesterol level, previous myocardial infarction, angina, heart failure, stroke, peripheral vascular disease, thromboembolic events, prior percutaneous coronary intervention, prior coronary artery bypass grafting, use of aspirin, B-Blocker, Lipid-lowering agent or ACE inhibitor.

## Discussion

### Summary of main findings

Multimorbidity is common in patients with cardiovascular disease in the community with 45% of patients also having chronic kidney disease, anaemia or both. Anaemia was relatively mild in this group with a mean haemoglobin of 10.90 g/dl (SD = 1.24) for women and a mean haemoglobin of 11.28 g/dl (SD = 1.80) for men. However, the presence of even mild anaemia with CKD was associated with a synergistic amplification of the risk of death. Patients with all three comorbidities had a greater than 4 times increased risk of death when compared with patients with CVD only.

### Strengths and limitations of this study

This study describes the prevalence of CKD and anaemia in a nationally representative community based cohort of patients with cardiovascular disease. To our knowledge, this is the first description of the comparative risks associated with anaemia and chronic kidney disease in a primary care population with established cardiovascular disease. The cohort comprised the complete cardiovascular population of a randomly selected sample of practices and despite the difficulties of detailed data collection in primary care very few patients were lost to follow-up (2.6%). However, our study had a number of limitations. Firstly, the cohort was not formed as a de novo population with multimorbidity but rather a cardiovascular population in which multimorbidity was then identified. Secondly, although level of multimorbidity appears to discriminate prognosis between patients with established cardiovascular disease, it is difficult to identify the different elements of this association as much of chronic kidney disease and anaemia may have their origin in cardiovascular disease. Thirdly, the study sample was overwhelmingly Caucasian and the prevalence of chronic kidney disease in this study may underestimate the prevalence in other ethnic groups. Fourthly, the sample sizes of the patient groups with anaemia were small (*n *= 38 and *n *= 37) in comparison to the other patient groups used in this study. Finally, lack of availability of serum creatinine and full blood count data in the community setting excluded some patients from the study and shortened follow-up in others. This reflects the limitation in data-availability which can hamper chronic disease management and indeed research within primary care generally.

### Comparison with existing literature

Population surveys suggest that 30% of adults suffer from more than one chronic health problem [20]. In the US, the prevalence of multimorbidity is estimated at 65% in those over 65 years of age [21]. There is a large discrepancy between the prevalence of multimorbidity in the population and the number of research studies devoted to it, especially in primary care [22]. The one year mortality of elderly patients with moderate chronic kidney disease (serum creatinine 220-343 μmol/L) after myocardial infarction is nearly tripled compared to those with normal renal function (66% vs. 24%) [23]. Similarly, survival is noticeably reduced in patients with anaemia both 1 month [24] and 1 year [25] after myocardial infarction. The current study confirms that in patients with CVD both CKD and anaemia have an additive effect in increasing risk of death.

The current data are consistent with what has been demonstrated elsewhere in patients with chronic kidney disease -- that anaemia is strongly associated with both cardiovascular disease [26,27] and adverse clinical outcomes including mortality [28-30]. The cumulative effect of multimorbidity in CVD and CKD has also been demonstrated with diabetic patients [16] and it is suspected that diabetes would play a further additive role on negative clinical outcomes in patients with highest levels of comorbidity.

### Implications for future research and clinical practice

The absence of national or indeed international guidelines for the management of patients with multimorbidity highlights the gulf which exists between the complexity of disease and the reductionism inherent in disease-specific guideline development. Specific to the comorbidities involved in this study, some authors of cardiorenal anaemia syndrome literature hypothesize that the pathophysiological processes may differ between anaemia and CKD when compared with anaemia and heart failure [10]. These differences may account for discrepancies that have been shown in the results from large multi-centre randomized trials and smaller trials of anaemia correction therapy. Many of the larger trials with CKD patients demonstrated no improvement with anaemia correction therapy [31-33], whereas smaller studies were able to demonstrate that anaemia correction improved NYHA class and renal function in patients with heart failure [34,35]. It is also clear that recent studies involving patients with CKD [3,33] or cancer with anaemia have raised concerns about potential harm from aggressive anaemia correction. There are currently two large ongoing trials that are attempting to resolve the divergence in the results of anaemia therapy in CKD and patients with heart failure [36,37]. There is a great deal of uncertainty that exists surrounding the management of anaemia in patients with both chronic kidney disease and cardiovascular disease. Although targets for haemoglobin levels in patients with CKD have been confirmed [38,39] anaemia correction therapy may not be commonly practiced in light of discrepancies between the results discussed above. It may be due to the uncertainty of target haemoglobin levels in multimorbidity patients that anaemia diagnosis and treatment is surprisingly low in CKD patients [40]. It is also possible that primary care physicians are prioritizing treatment of CVD over CKD and anaemia in this population [41]. Patients with these co-morbidities may also suffer from late referral to secondary care [42]. We have demonstrated that even milder levels of anaemia may be associated with negative health outcomes in patients with CKD and/or CVD.

## Conclusion

In patients with cardiovascular disease, the presence of chronic kidney disease carries an increased mortality risk which increases in an additive way with the addition of anaemia. These results suggest that early primary care diagnosis and management of this high risk group may be worthwhile. In order to develop effective management strategies, however, innovative approaches to the study of this population are required and due to the diversity of patients and medical conditions encountered, primary care offers an ideal setting for this to take place.

## Ethical Approval

Ethical approval was granted by the research ethics committee of the Irish College of General Practitioners (Protocol No: REC0904-4).

## Competing interests

AWM has received funding from Pfizer to support educational meetings for general practitioners who teach medical students from the Department of General Practice at NUI, Galway. LG and DR have received an honorarium from Roche laboratories for contribution to the development of CKD guidelines for primary care. JA and JN declare no conflict of interest.

## Authors' contributions

JA, LG (guarantor), DR, AWM, and JN contributed to study conception and design. LG was responsible for the acquisition of data while LG, AWM, DR, JN, AI and JA analysed the data and drafted the article. All authors revised the article and granted final approval to the version submitted for publication.

## Pre-publication history

The pre-publication history for this paper can be accessed here:

http://www.biomedcentral.com/1471-2261/9/51/prepub
